# Mathematical Model of Solid Food Pasteurization by Ohmic Heating: Influence of Process Parameters

**DOI:** 10.1155/2014/236437

**Published:** 2014-01-19

**Authors:** Francesco Marra

**Affiliations:** Dipartimento di Ingegneria Industriale, Università degli Studi di Salerno, Via Giovanni Paolo II 132, 84084 Fisciano, Italy

## Abstract

Pasteurization of a solid food undergoing ohmic heating has been analysed by means of a mathematical model, involving the simultaneous solution of Laplace's equation, which describes the distribution of electrical potential within a food, the heat transfer equation, using a source term involving the displacement of electrical potential, the kinetics of inactivation of microorganisms likely to be contaminating the product. In the model, thermophysical and electrical properties as function of temperature are used. Previous works have shown the occurrence of heat loss from food products to the external environment during ohmic heating. The current model predicts that, when temperature gradients are established in the proximity of the outer ohmic cell surface, more cold areas are present at junctions of electrodes with lateral sample surface. For these reasons, colder external shells are the critical areas to be monitored, instead of internal points (typically geometrical center) as in classical pure conductive heat transfer. Analysis is carried out in order to understand the influence of pasteurisation process parameters on this temperature distribution. A successful model helps to improve understanding of these processing phenomenon, which in turn will help to reduce the magnitude of the temperature differential within the product and ultimately provide a more uniformly pasteurized product.

## 1. Introduction

Recent trends show that development of new technologies for thermal food treatment are widely applied, from thawing [1] to combined drying [2, 3], to pasteurization and cooking [4, 5]. Electroheating processes, such as microwave, radiofrequency, or ohmic heating, can help industry to develop faster and more efficient thermal processes, including inactivation of microorganism potentially affecting foods, that is an important industrial application using heat as principle responsible of microbial inactivation [6–8].

Among electroheating processes, ohmic heating consists of the direct passage of electric current through the product. The permanent motion of electrical charges creates heat in the product in agreement with Joule's law. According to this principle, ohmic technology could be considered as purely bulk heating. Nowadays, a large number of potential future applications exist for ohmic heating: blanching, evaporation, dehydration, fermentation, and pasteurization/sterilization of liquid or liquid-containing particulates [7].

The main advantages of ohmic processing are the rapid and relatively uniform heating achieved [5]. This is expected to reduce the total thermal abuse to the product in comparison to conventional heating, where time must be allowed for heat penetration to occur to the center of a material and particulates heat slower than the fluid phase of a food. While some evidence exists for nonthermal effects of ohmic heating, the principal mechanisms of microbial inactivation in ohmic heating are thermal in nature. The applicability is limited to foods with sufficient conductivity: often, in order to confer the optimal electrical conductivity, solid and particulate foods are pretreated in brine solutions. Ohmic processing of liquids is industrially applied; ohmic heating of solid foods has not yet led to commercial applications although few works indicate that this technology shows great promise [9–11]. Being fundamentally a thermal-based process, temperature and time are the principal critical process factors and, as in conventional thermal processes, the key problem is locating the spot or the areas of minimum thermal treatment, as in other electroheating applications [12, 13]. Mathematical modelling is an invaluable aid in the development, understanding, and validation of these emerging thermal technologies [14]. As in any thermal process, the development of a mathematical model has to be addressed to identify possible hot and cold spots, to quantify heat losses, and to evaluate the influence of key variables such as electrical field strength and sample conductivity.

**Figure 1:**
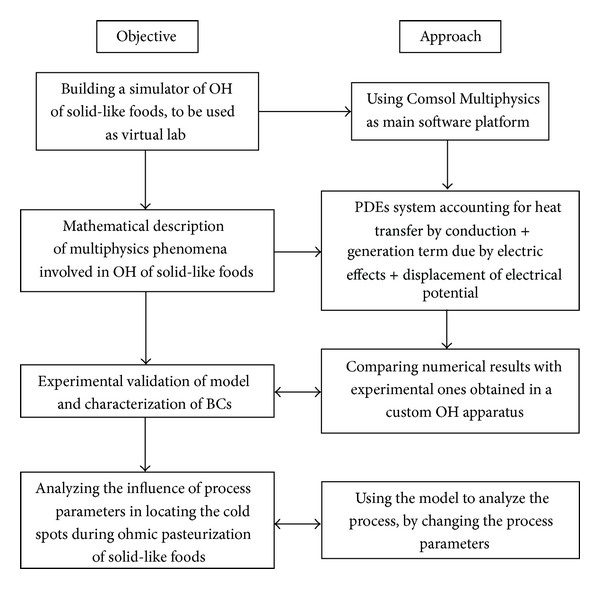
Flow-sheet of mutual connections existing between the objective and the approach used in the present work.

The goal of this paper is to build a simulator, to be used as virtual lab for ohmic heating tests and for analyzing the influence of process parameters in locating the cold spot or areas during ohmic heating of solid-like foods, using a model developed and validated in a previous work [10]. The mutual connections existing between the objective and the approach used are summarized in Figure 1.

## 2. Material and Methods

### 2.1. Mathematical Modelling

#### 2.1.1. Governing Equations

Modelling ohmic pasteurization involves simultaneous solution of

1. Laplace's equation, which describes the distribution of electrical potential within a food,
2. heat transfer equation, using a source term involving the displacement of electrical potential,
3. kinetics of inactivation of microorganisms likely to be contaminating the product.

In the model, a cylinder filled with mashed potatoes is considered as sample. The choice of mashed potatoes as model food is due to its highly uniform nature. Samples for model validation were prepared according to the protocol reported by Marra et al. [10].

**Figure 2:**
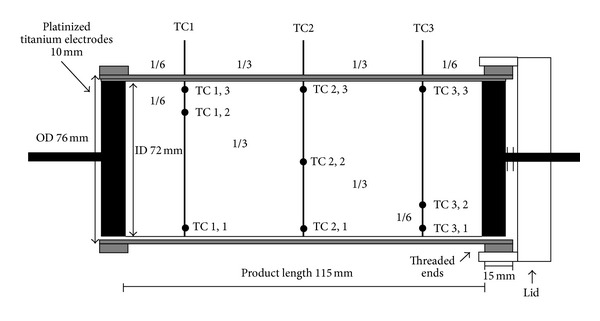
Sketch of OH cell used in the experimental validation of the model, including 9 thermoprobes' positions [10].

Configuration of heating system is reported in Figure 2. The sample is contained in a hollow cylinder made of teflon, that is held by the dark blue electrodes.

According to quasistatic approach, the electrical potential distribution within the sample can be computed using the following Laplace equation:

(1) $$\begin{array}{c} \nabla \cdot \sigma \nabla V=0. \end{array}$$

Electrical potential distribution and, then, electrical conduction generate into the product a certain density of power, as described by the following equation:

(2) $$\begin{array}{c} Q_{\text{GEN}}=\sigma {\left|\nabla V\right|}^{2}, \end{array}$$

where *σ* is the electrical conductivity, and |∇*V*| represents the modulus of the gradient of electrical potential.

The heat transfer occurring during the process is described by the classical unsteady state heat equation by conduction plus the generation term described by (2), as

(3) $$\begin{array}{c} \rho Cp\frac{\partial T}{\partial t}=\nabla \cdot \lambda \nabla T+Q_{\text{GEN}}, \end{array}$$

where *T* is the temperature within the sample, *t* is the process time, *λ* is the thermal conductivity, *ρ* is the density, *Cp* is the heat capacity, and *Q*_GEN_ represents the ohmic power source, as in (2).

Since the electrical conductivity *σ* is a function of temperature, (1) and (3) are strictly related to each other and must be solved simultaneously [15].

Once temperature distribution is known, the coldest point or areas can be determined and, in their correspondence, lethality of microorganisms can be calculated. In fact, the decay of microbial population *N* with the processing time follows a first-order rate defined in the following equation:

(4) $$\begin{array}{c} \frac{dN}{dt}=\frac{2.303}{D}N, \end{array}$$

where the decimal decay *D* value is defined as

(5) $$\begin{array}{c} D=D_{r}10^{\left(T_{\text{ref}}-T\right)/z} \end{array}$$

being a measure of the heat resistance of a microorganism: it represents the time (in minutes) at a given temperature required to destroy 1 log cycle (90%) of the target microorganism. Of course, in an actual process, all others that are less heat tolerant are destroyed to a greater extent. For example, a *D* value at 100°C of 1 minute means that, for each minute of processing at 100°C, the bacteria population of the target microorganism will be reduced by 90%.

Imposing an initial microbial population *N*_0_ at time *t* = 0, (4) is integrated to give

(6) $$\begin{array}{c} \log\frac{N_{0}}{N}=\frac{1}{D_{r}}\overset{t_{f}}{\underset{0}{\int }}10^{\left(T-T_{\text{ref}}\right)/z}dt, \end{array}$$

where *T* is the cold spot temperature at any time *t*, *T*_ref_ is the reference temperature of the target microorganism, and *z* is the corresponding *z*-value; the integral ∫_0_^*t*_*f*_^10^(*T*−*T*_ref_)/*z*^ *dt* is also known as sterility factor *F*, that—for a given heating process of food—represents the accumulated lethality and it is the number of minutes required to kill a known population of microorganisms in the processed food under specified conditions. Traditionally, *F* value is usually set at 12*D* values to give a theoretical 12 log cycle reduction of the most heat-resistant species of mesophilic spores in a can of food (i.e., if there were 10^6^ spores of a species of spore in a can of food and a 12*D* process was given, the initial 10^6^ spores would be reduced to a theoretical 10^−6^ living spores per can, or again in theory, one living spore per 10^6^ cans of product (one spore per one hundred million cans)).

#### 2.1.2. Initial and Boundary Conditions

Laplace's equation (1) needs only boundary conditions to be solved, being a stationary-state equation; the heat transfer equation (2) needs initial condition and boundary conditions.

For Laplace's equation, the following boundary conditions were assumed: an applied voltage between the two electrodes and a complete electrical insulation of the lateral external sample surface. A fixed applied voltage of 100 V has been considered in this work.

Prior to commencing ohmic heating, it is assumed that the entire sample is at a uniform temperature *T*_0_. As boundary conditions for the heat transfer equation, two different cases were considered: the first one assumed that all the sample are thermally insulated; the second one assumed a general external heat transfer (Newton cooling law) given by

(7) $$\begin{array}{c} q_{\text{ext}}=U\left(T-T_{\inf}\right), \end{array}$$

where *U* is an overall heat transfer coefficient, that takes into account any possible composite resistance such as multilayers around the ohmic cell and *T*_inf_ is the external environment temperature. About the role of overall heat transfer coefficient, two conditions were considered: a thermally insulated condition and a condition where heat lost toward the external environment was considered.

The thermally insulated case represents the best process condition, given that no heat is lost toward the external environment. The second case represents one of possible conditions when heat is lost toward the external environment: particularly, 5 < *U* < 10 W m^−2^ K^−1^ corresponds to the typical range of values for overall heat transfer coefficient under conditions similar to the ones considered in this work [16]. An experimental validation of the model suggested that the value of *U* is 5 W m^−2^ K^−1^, being the best fitting value provided by experimental validation of model [10].

### 2.2. Sample Properties and Parameters

As sample food, mashed potatoes were considered. Thermophysical properties (thermal conductivity and volumetric heat capacity) of sample were measured using a KD 2 probe (Decagon Devices, Pullman, WA, USA); its electrical conductivity was measured by means of a set-up described by Olivera et al. [17].

As target microorganism contaminating the sample, *Escherichia Coli* O_157_ : H_7_ (*E. Coli*), has been considered. *E. Coli* is characterized by a *z*-value of 10°C [12].

### 2.3. Numerical Solution

The set of equations introduced above, with their relative initial and boundary conditions, were solved by means of a commercial software, Comsol 3.4 (Comsol AB, Stockholm, Sweden), running on a personal computer, equipped with two Intel Xeon CPUs (Intel, Santa Clara, CA, USA), at 2.00 GHz, with 4 Gb of RAM, running under Windows XP Professional (Microsoft, Redmont, WA, USA).

An implicit time-stepping scheme was used to solve time-dependent problems: at each time step, the software solved a possibly nonlinear system of equations. The nonlinear system was solved using a Newtonian iteration. An arbitrary linear system solver was then used for the final resulting systems (Comsol 3.4 User Guide). For the purposes of this research, a direct linear system solver (UMFPACK) was used. Relative tolerance was set at 1 × 10^−2^, whereas absolute tolerance was set at 1 × 10^−3^.

Numerical tests were performed with different mesh parameters in order to evaluate the simulation results and to find the best mesh settings. In Table 1 is reported the mesh set providing the best spatial resolution for the considered domain and for which the solution was found to be independent of the grid size.

### 2.4. Validation of Temperature Distribution

In order to validate the temperature distribution provided by the model solution, an experimental set-up has been prepared using the same material (mashed potatoes) and replicating the same procedures described in Marra et al. [10].

## 3. Results 

Thermophysical properties and electrical conductivity were measured and the obtained data were reported in Table 2. These values were then introduced in the mathematical model as vectors depending on the local temperature value, in order to account for the variability of properties in each point of the food substrate.

**Figure 3:**
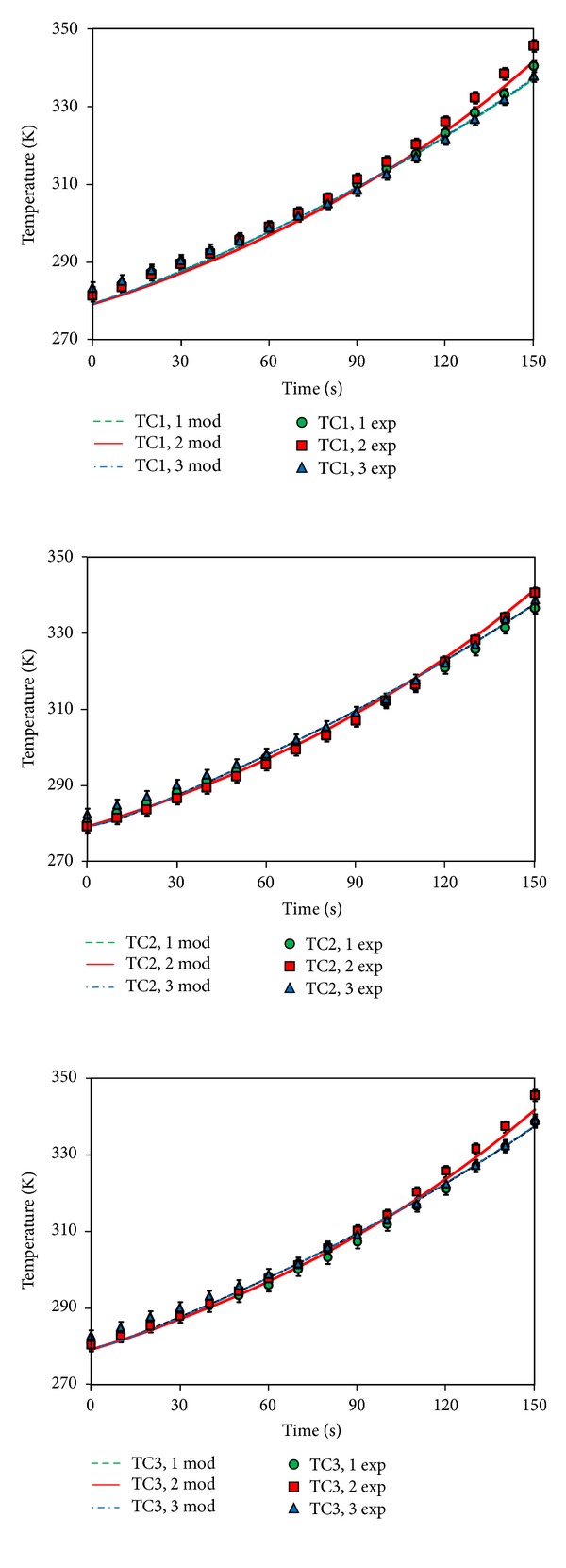
Comparison among experimental results (markers) and simulation results (lines) at 9 measurement points as in Figure 2. Total processing time: 150 seconds; applied voltage set point: 100 V; *T*_0_ = 279.15 K; *U* = 5** **W** **m^−2^ K^−1^; *T*_inf_ = 286.15 K.

The solution of the mathematical model described in the previous section provides the distribution, in the space and during the time, of temperature, as well as the reduction of the heat-resistant microorganisms. The validation of the model is shown in Figure 3, where the temperature values read at the nine measurement points are compared with the temperature evolution provided, at the same positions, by the solution of the mathematical model. The model results are referred to an overall heat exchange coefficient *U* = 5 W m^−2^ K. The agreement between the experimental data and the model ones is fairly acceptable; the only evidently less according data are referred to positions 1, 2 and 3, 2, at final processing time, where the measured values are about 7°C higher than the ones foreseen by the model.

Both experiments and model results confirmed results available in the literature, where it has been reported that—under ohmic heating—homogeneous solid-like foods were heated up evenly but temperature gradients were established in proximity of sample edge, suggesting that an accurate thermal insulation of the sample can result in a more efficient process [10, 18].

In order to understand the influence of some process parameters, such as the conditions (represented by coefficient *U* and external temperature *T*_inf_) of heat exchange with the external environment, the thermal diffusivity of the sample and its electrical conductivity, and the difference of potential applied between the electrodes, a sensitivity analysis was accomplished by varying each parameter and then reading the corresponding change in temperature and in accumulated lethality.

Furthermore, two special cases were considered between the electrodes for the runs with perfected insulated walls: (1) maintaining a constant applied voltage and (2) maintaining a constant heat generation. These two cases are very interesting because, while the sample temperature after 150 seconds is virtually the same, its time evolution (and so the lethality and the accumulated lethality) is different.

**Figure 4:**
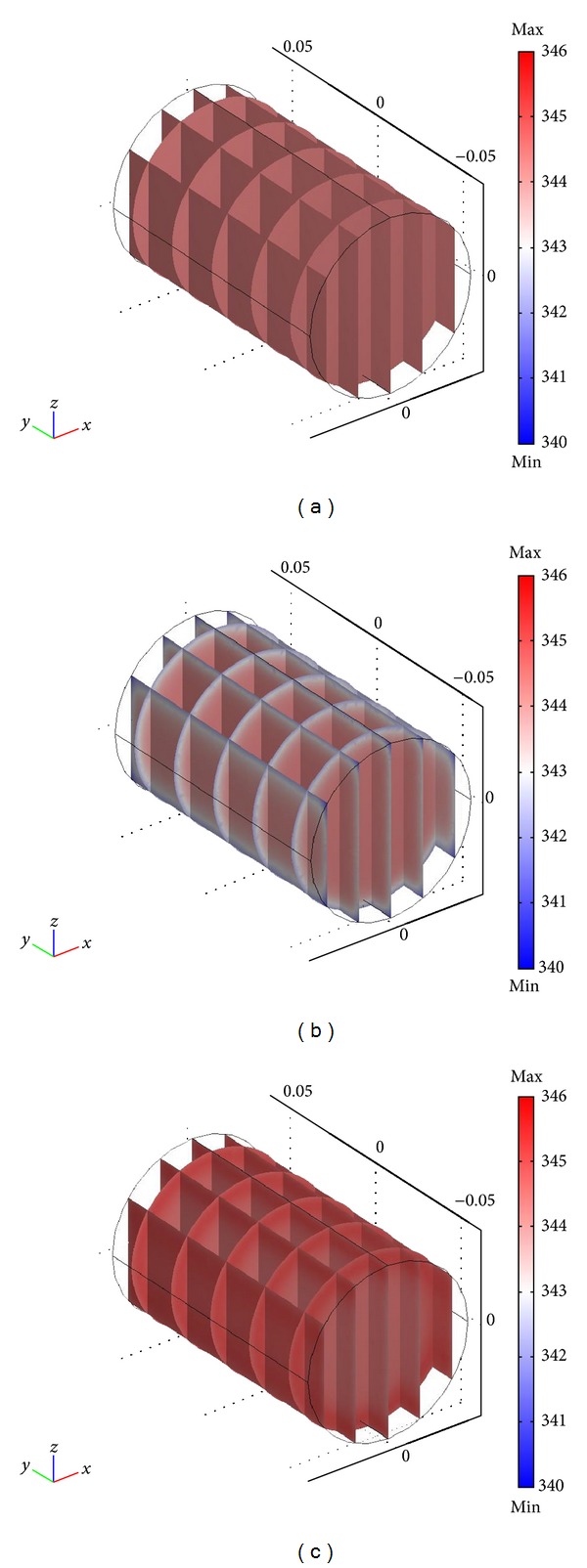
Slice plots representing temperature after 150 seconds of OH: applied voltage set point: 100 V; *T*_0_ = 279.15 K; *U* = 0** **W m^−2^ K^−1^ (a); *U* = 5 W m^−2^ K^−1^ ((b) and (c)) *T*_inf_ = 286.15 K (b) and *T*_inf_ = 314.15 K (c).

Different temperature distributions, reported as slice plots after 150 seconds of ohmic heating driven by a differential of electrical potential between electrodes of 100 V, are shown in Figure 4, where no external heat exchange (*U* = 0 W m^−2^ K^−1^, Figure 4(a)), best fitting value for convective heat exchange coefficient with cold external temperature (*U* = 5 W m^−2^ K^−1^; *T*_inf_ = 286.15 K, Figure 4(b)) and with warm external temperature (*U* = 5 W m^−2^ K^−1^; *T*_inf_ = 314.15 K, Figure 4(c)) was considered. The plots emphasize that an ideally insulated ohmic cell (Figure 4(a)) results in a uniform heating of the sample. In a similar case, it is almost impossible to identify, clearly, a colder area in the investigated domain; thus, the calculation of the *F*-value can be done in any point of the sample. When a low but effective heat convection is realized in proximity of the sample boundary (as in the validated case, when the heat transfer coefficient *U* is 5 W m^−2^ K^−1^), a temperature gradient develops in a sample layer in proximity of the boundaries (Figure 4(b)). In this case, the coldest area exists where the *F*-value has to be calculated. By keeping the hypothesis of low but effective heat convection realized in proximity of the sample boundary, but imposing a higher temperature of external air, a third case is considered, when the loss of heat from the sample toward the external environment is lowered by the lowered driving force of the Newton cooling law (7). Results referring to this last case (Figure 4(c)) show that the reduced heat transfer from the sample toward the external environment results in a warmer external shell, so that the coldest area of the sample is located on the axis.

In order to analyze the process both in terms of temperature distribution and time required to attain the desired reduction (12*D*) of target microorganism, seven cases were considered, differing one with respect to the others, because of the values of *U* and *T*_inf_, and because of different electrical conductivity values. The characteristic parameters of the seven considered cases are the following:

C1: *U* = 0 W m^−2^ K^−1^;
C2: *U* = 0 W m^−2^ K^−1^ and electrical conductivity +10% with respect to S1;
C3: *U* = 0 W m^−2^ K^−1^ and electrical conductivity −10% with respect to S1;
C4: *U* = 5 W m^−2^ K^−1^, with electrical conductivity as in S1 and *T*_inf_ = 286.15 K;
C5: *U* = 5 W m^−2^ K^−1^, with electrical conductivity as in S1 and *T*_inf_ = 314 K;
C6: *U* = 15 W m^−2^ K^−1^, with electrical conductivity as in S1 and *T*_inf_ = 286.15 K;
C7: *U* = 15 W m^−2^ K^−1^, with electrical conductivity as in S1 and *T*_inf_ = 314 K.

**Figure 5:**
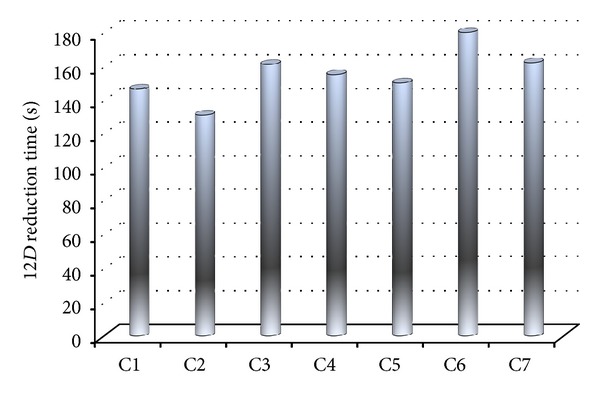
Time required to attain a 12*D* reduction of target microorganism. C1: *U* = 0 W m^−2^ K^−1^; C2: *U* = 0 W m^−2^ K^−1^ and electrical conductivity +10% with respect to S1; C3: *U* = 0** **W m^−2^ K^−1^ and electrical conductivity −10% with respect to S1; C4: *U* = 5 W m^−2^ K^−1^, with electrical conductivity as in S1 and *T*_inf_ = 286.15 K; C5: *U* = 5** **W** **m^−2^ K^−1^, with electrical conductivity as in S1 and *T*_inf_ = 314 K; C6: *U* = 15 W m^−2^ K^−1^, with electrical conductivity as in S1 and *T*_inf_ = 286.15 K; C7: *U* = 15 W** **m^−2^ K^−1^, with electrical conductivity as in S1 and *T*_inf_ = 314 K.

The results in terms of time required to attain a 12*D* reduction of the target microorganism (*E. Coli*) are reported in Figure 5. Please note that cases C2 and C3 are meant to understand the role played by the electrical conductivity in the attainment of 12*D* reduction of the target microorganism. An increase of electrical conductivity of 10% allows lowering the 12*D* reduction time; the opposite happens when the electrical conductivity increases. Warmer air surrounding the sample (C5 and C7) provides shorter treatment times with respect to cases where colder air is around the sample (cases C4 and C6). As results show, the case C6 is the less favorite case (180 seconds are needed to obtain a 12*D* reduction of the contaminant microorganism).

**Figure 6:**
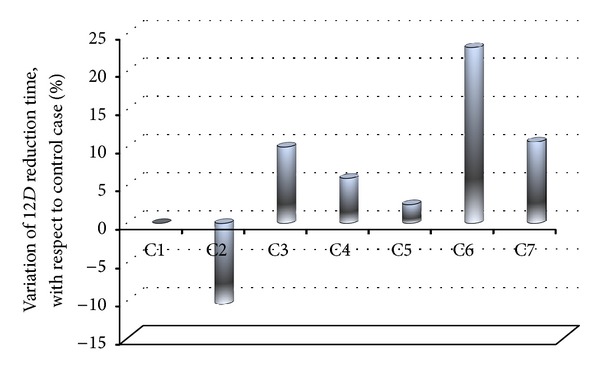
Variation of time required to attain a 12*D* reduction of target microorganism with respect to the control case, C1. C1: *U* = 0 W m^−2^ K^−1^, control case; C2: *U* = 0 W m^−2^ K^−1^ and electrical conductivity +10% with respect to S1; C3: *U* = 0 W m^−2^ K^−1^ and electrical conductivity −10% with respect to S1; C4: *U* = 5 W m^−2^ K^−1^, with electrical conductivity as in S1 and *T*_inf_ = 286.15 K; C5: *U* = 5 W m^−2^ K^−1^, with electrical conductivity as in S1 and *T*_inf_ = 314 K; C6: *U* = 15 W m^−2^ K^−1^, with electrical conductivity as in S1 and *T*_inf_ = 286.15 K; C7: *U* = 15 W m^−2^ K^−1^, with electrical conductivity as in S1 and *T*_inf_ = 314 K.

It is possible to verify how the 12*D* reduction time changes with respect to case C1, that is taken as control case (Figure 6). With respect to control case, an increase of 10% in the electrical conductivity causes a decreasing (of about 10%) of the 12*D* reduction time. The opposite happens when a decreasing of 10% in electrical conductivity is considered. The higher variation is expected when case C6 is compared with the control case. Results then show how important is the electrical conductivity of the sample to be heated by ohmic effects and how important is to have accurate measurement of this property, given that a small error in the electrical conductivity determination will result in a big error in phase of designing and validation of a ohmic based heater.

About the role played by the external heat convection, the computation of decimal reduction of microorganism potentially contaminating the food demonstrated that a slight heat loss does not change the time required to attain a 12*D* reduction of target microorganism. In any case, heat losses toward the external environment must be kept within a narrow range, to avoid—as for case C6—prolonged processing time.

## 4. Conclusions

Numerical analysis of ohmic heating of solid foods allowed understanding the role played by some process parameters in temperature distribution within the product and achievement of required time for pasteurization (12*D* reduction of *E. Coli*). Results showed that, according to the appropriate boundary condition, external areas of the product present the slower heating and have to be monitored in terms of target microorganism lethality. Pasteurization time is particularly influenced by the sample electrical conductivity, since the generation term appearing in the heat equation is predominant with respect to thermal conduction.

Mathematical modeling is a useful tool for designing an ohmic heating unit for pasteurization of solid foods and virtual experiments can provide important information for developing an effective control system for this process

## Figures and Tables

**Table 1:** Benchmarked mesh set.

Mesh elements			Degree of freedom
Tetrahedrons	Boundary	Edges	
10217	1396	100	30170

**Table 2:** Measured values of thermal conductivity (*λ*), volumetric heat capacity (VHC), and electrical conductivity (*σ*).

Temperature (°C)	**λ** (W/(m K))	VHC (MJ/(m^3^ K))	**σ** (S/m)
5	0.409	3.339	1.228
25	0.451	3.383	1.991
45	0.490	3.441	2.756
65	0.532	3.496	3.514
85	0.571	3.589	4.278
